# Genome-Wide Characterization and Comparative Analysis of MYB Transcription Factors in *Ganoderma* Species

**DOI:** 10.1534/g3.120.401372

**Published:** 2020-05-29

**Authors:** Lining Wang, Qinghua Huang, Liulian Zhang, Qingfu Wang, Lei Liang, Baosheng Liao

**Affiliations:** * Guangdong Provincial Engineering Laboratory of Biomass High Value Utilization, Guangzhou Key Laboratory of Biomass Comprehensive Utilization, Guangdong Bioengineering Institute (Guangzhou Sugarcane Industry Research Institute), Guangdong Academy of Sciences, Guangzhou, 510316, Guangdong Province, China and; ^‡^ Institute of Chinese Materia Medica, China Academy of Chinese Medical Sciences, Beijing, 100700, China

**Keywords:** *Ganoderma*, *MYB* gene family, genome-wide analysis, comparative analysis

## Abstract

Numerous studies in plants have shown the vital roles of MYB transcription factors in signal transduction, developmental regulation, biotic/abiotic stress responses and secondary metabolism regulation. However, less is known about the functions of *MYBs* in *Ganoderma*. In this study, five medicinal macrofungi of genus *Ganoderma* were subjected to a genome-wide comparative analysis of *MYB* genes. A total of 75 *MYB* genes were identified and classified into four types: 1R-MYBs (52), 2R-MYBs (19), 3R-MYBs (2) and 4R-MYBs (2). Gene structure analysis revealed varying exon numbers (3-14) and intron lengths (7-1058 bp), and noncanonical GC-AG introns were detected in *G. lucidum* and *G. sinense*. In a phylogenetic analysis, 69 out of 75 *MYB* genes were clustered into 15 subgroups, and both single-copy orthologous genes and duplicated genes were identified. The promoters of the *MYB* genes harbored multiple *cis*-elements, and specific genes were co-expressed with the *G. lucidum MYB* genes, indicating the potential roles of these *MYB* genes in stress response, development and metabolism. This comprehensive and systematic study of MYB family members provides a reference and solid foundation for further functional analysis of *MYB* genes in *Ganoderma* species.

*Ganoderma* species, which are widely distributed around the world, are important macrofungi belonging to Ganodermataceae (Polyporales, Basidiomycota). The most common pharmacological activities of *Ganoderma* species are antitumour, antioxidant, and antimicrobial activities, and at least 19 *Ganoderma* species have been studied for their medicinal functions in China to date (Dai *et al.* 2009; Yi *et al.* 2015; Basnet *et al.* 2017; Wu *et al.* 2019a). Polysaccharides (Cör *et al.* 2018; Ma *et al.* 2018) and triterpenes (Liang *et al.* 2019; Wahba *et al.* 2019) with vital medicinal functions have drawn the attention of researchers. In addition, great progress has been made in the classification/identification (Liao *et al.* 2015), genetic diversity (Midot *et al.* 2019), and fruiting body development (Sudheer *et al.* 2018) of *Ganoderma* species.

The MYB (myeloblastosis) transcription factor family is one of the largest transcription factor families and is widely distributed in eukaryotic organisms (Riechmann *et al.* 2000). The MYB domain contains 1 to 4 imperfect repeats that consist of approximately 50 amino acids (aa), and the repeat is characterized by conserved and regularly spaced tryptophans, which form a hydrophobic core to maintain the helix-turn-helix (HTH) secondary structure of the repeat (Sakura *et al.* 1989; Ogata *et al.* 1994). Accordingly, the MYB gene family is divided into four subfamilies, including 1R-MYB, 2R-MYB, 3R-MYB, and 4R-MYB (Ogata *et al.* 1994). Since the first identification of *MYB* genes from avian myeloblastosis virus (Klempnauer *et al.* 1982) and from plants (Paz‐Ares *et al.* 1987), the function of *MYB* genes has been widely studied. Abundant evidence in plants has confirmed the diverse roles of *MYB* genes in signal transduction, developmental regulation, biotic and abiotic stress responses, disease resistance, and secondary metabolism regulation (An *et al.* 2019; Lee & Seo 2019; Qing *et al.* 2019; Wu *et al.* 2019b; He *et al.* 2020; Ohno *et al.* 2020). Similarly, a few studies have shown that *MYB* genes participate in stress response and developmental regulation in fungi (Valsecchi *et al.* 2017; Wang *et al.* 2018; Li *et al.* 2019).

Taking advantage of the numerous available genomes, comprehensive information regarding number, classification, gene structure, evolution, and expression pattern of *MYB* genes has been widely analyzed, especially in plants and fungi, such as *Arabidopsis thaliana* (Chen *et al.* 2006), *Gossypium hirsutum* (Salih *et al.* 2016), *Phyllostachys edulis* (Yang *et al.* 2019), *Pleurotus ostreatus* (Wang *et al.* 2018), and *Ophiocordyceps sinensis* (Li *et al.* 2019). Several genomes of *Ganoderma* species are currently available (Chen *et al.* 2012; Zhu *et al.* 2015); nevertheless, little is known about the *MYB* genes of *Ganoderma* species. Given the critical roles of *MYB* genes in eukaryotic organisms, exploring *MYB* genes among *Ganoderma* species will contribute to a better understanding of the specific structural and functional characteristics of macrofungi.

In this study, the *MYB* gene family members in *Ganoderma* species, including *G. australe*, *G. boninense*, *G. lucidum*, *G. sinense* and *G. tsugae*, were extensively characterized. Gene numbers, sequence features, gene structures, genetic relationships and promoter *cis*-elements were analyzed and compared among these five species. An additional co-expression analysis of *MYB* genes was performed in *G. lucidum*. These investigations provide fundamental information about the *Ganoderma MYB* gene family and will facilitate gene function elucidation and molecular assisted breeding of *Ganoderma*.

## Materials And Methods

### Genome sequences and gene sets acquisition

The genome sequences of five *Ganoderma* species downloaded from NCBI BioProjects were used in this study*: G. australe* (PRJNA476322), *G. boninense* (PRJNA421251), *G. lucidum* (PRJNA71455), *G. sinense* (PRJNA42807) and *G. tsugae* (PRJNA445345). Gene annotation results of *G. sinense* were downloaded from GenBank (https://www.ncbi.nlm.nih.gov/assembly/GCA_002760635.1/). For other genomes, gene models were predicted using Maker pipeline (Campbell *et al.* 2014) with *G. sinense* CDS sequences as EST evidence and UniProt protein sequences as protein evidence.

### Identification and classification of MYB genes in Ganoderma species

All the annotated proteins in the five *Ganoderma* genomes were searched against to PFAM database (Pfam 32.0) with PfamScan (evalue ≤ 1e-5) (http://www.ebi.ac.uk/Tools/pfa/pfamscan). Genes with hits to PFAM ID PF00249.30, PF08914.10, PF11831.7, PF12776.6, PF13921.5, PF15963.4 and PF16282.4, and with out PF04433.16 and PF16495.4 (SWIRM/SWIRM-associated) were considered as candidate *MYB* genes. Then, *MYB* genes were viewed and corrected on Apollo (Dunn *et al.* 2019) browser: 1) each of the candidate *MYB* genes of *G. lucidum*, *G. sinense* and *G. boninense* was manually corrected based on the alignments between genome sequences and corresponding transcriptome data (PRJNA269646, PRJNA514399, PRJNA71455, PRJNA374969, PRJNA574544, PRJNA42807) with HISAT2 (Pertea *et al.* 2016); 2) the candidate *MYB* genes of *G. australe* and *G. tsugae* were corrected based on the alignments with their homologous *MYB* genes in the other three species with BLASTP (protein sequences) and BLASTN (coding sequences) (Camacho *et al.* 2009). All the above corrected sequences were searched against the five *Ganoderma* genomes and gene sets using both BLASTN and TBLASTN (Camacho *et al.* 2009) (query length coverage ≥ 60%, sequence identity ≥ 60%) to avoid missing false-negative identification. Finally, all the *MYB* genes were reconfirmed by PfamScan to rule out false-positive results. The positions and numbers of the MYB domains and other domains were detected by PfamScan, and the *MYB* genes were classified into 1R-, 2R-, 3R- and 4R-MYB groups. A synteny analysis of the *MYB* genes was conducted between the two most complete genomes, *G. lucidum* and G. *sinense*, using MCSCAN (Tang *et al.* 2008).

### Sequence information, gene structure, and phylogenetic analysis of MYB genes

The theoretical isoelectric point (pI) and molecular weight (MW) were computed using the online Compute pI/Mw tool (http://web.expasy.org/compute_pi/). The subcellular localization of each MYB protein was predicted by the online WoLF PSORT tool (https://wolfpsort.hgc.jp/). Conserved MYB domain sequences were analyzed and displayed by WebLogo (http://weblogo.berkeley.edu/logo.cgi). The gene structures of the *MYB* genes were investigated using TBtools based on the genomic features defined in the gff file (Chen *et al.* 2018). The distribution of intron length was drawn by ggplot2 (Wickham 2016) in R.

A multiple sequence alignment of the *Ganoderma MYB* genes was performed using ClustalOmega based on full-length protein sequences (Sievers *et al.* 2011), and a maximal likelihood (ML) phylogenetic tree was constructed using RAxML (Stamatakis 2014) with 1000 bootstrap replicates and plotted by Interactive Tree Of Life (iTOL) (Letunic & Bork 2019). In addition, an ML tree containing an additional 124 MYB protein sequences (including fungi, plants and animals) was built using the same method (Supplementary Table S1). The orthologous relationships of *MYB* genes in *Ganoderma* were determined using OrthoMCL v2.0.9 (Li *et al.* 2003). The single copy orthologous *MYB* genes were defined as follows: clustered in phylogenetic tree and in ortho-groups from OrthoMCL, and only one gene in each species.

### Analysis of promoter regions of Ganoderma species

The promoter regions of the *MYB* genes, which were defined as the 2000 bp upstream of the transcript start, were extracted and used in the following analysis. *GtMYB14* was not included in this analysis, as this gene starts at the beginning of the genome sequence. The *cis*-elements in the promoter regions were detected using PlantCARE (http://bioinformatics.psb.ugent.be/webtools/plantcare/html/). A hierarchical clustering analysis of *cis*-elements in *Ganoderma MYB* genes was performed using the pheatmap package (https://cran.r-project.org/web/packages/pheatmap/) in R.

### Co-expression of MYB genes in G. lucidum

Sixteen RNA-seq datasets of *G. lucidum* were downloaded from NCBI: SRR364264, SRR10199514, SRR10199515, SRR10199516, SRR10199517, SRR10199518, SRR10199523, SRR10199520, SRR10199521, SRR10199519, SRR10199522, SRR364265, SRR364266, SRR5261648, SRR5261647, SRR5261649. A co-expression network was built for the *MYB* genes of *G. lucidum* (*GlMYBs*) by the Weighted Gene Co-expression Network Analysis (WGCNA) package (Langfelder & Horvath 2008) in R. The genes in modules containing *GlMYBs* were extracted and subjected to GO classification using eggnog-mapper (Huerta-Cepas *et al.* 2019).

### Data availability

The necessary information of public data used in this study are present within the article. Data S1 shows sequences of 75 *Ganoderma MYB* genes. Data S2 shows GO classifications of genes related with *GlMYBs*. Table S1 shows accession numbers of 124 MYB proteins of other organisms used in the phylogenetic study. Table S2 shows comprehensive information of *MYB* genes identified in five *Ganoderma* species. Table S3 shows orthologous groups identified by OrthoMCL. Table S4 shows distribution of intron lengths. Table S5 shows length of MYB domains. Table S6 shows PfamScan results of 75 *MYB* genes in *Ganoderma* species. Figure S1 shows protein sequence alignment between *GaMYB10* and *GaMYB12*. Figure S2 shows protein sequence alignment between *GaMYB05* and *GaMYB11*. Figure S3 shows protein sequence alignment among *GtMYB07*, *GtMYB10*, *GtMYB11* and *GtMYB09*. Figure S4 shows phylogenetic relationships of *MYB* genes among *Ganoderma* species and other organisms. Figure S5 shows sequence logos of MYB domains in different subgroups. Figure S6 shows co-expression modules in *G. lucidum*. Supplemental material available at figshare: https://doi.org/10.25387/g3.12286277.

## Results

### Identification, classification and sequence information of MYB genes in Ganoderma species

**Figure 1:**
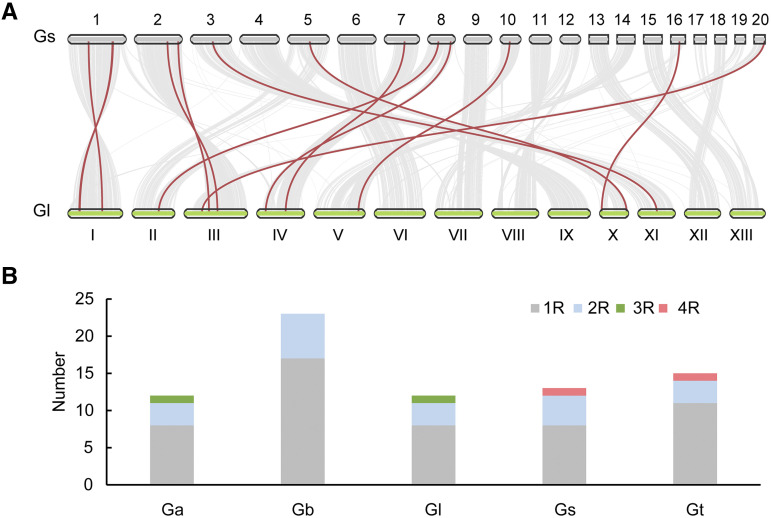
Synteny analysis of *MYB* genes in *G. lucidum* and G. *sinense* (A) and distribution of *MYB* genes in five *Ganoderma* species (B). Numbers 1-20 represent the 20 largest scaffolds of *G. sinense*, and I-XIII represent the 13 chromosomes of *G. lucidum*. The gray lines in the background represent collinear blocks between *G. lucidum* and G. *sinense*, while the red lines represent syntenic *MYB* gene pairs. Ga, *G. australe*; Gb, *G. boninense*; Gl, *G. lucidum*; Gs, *G. sinense*; Gt, *G. tsugae*.

Overall, 75 *MYB* genes were identified in *G. australe* (*GaMYB01-12*), *G. boninense* (*GbMYB01-23*), *G. lucidum* (*GlMYB01-12*), *G. sinense* (*GsMYB01-13*) and *G. tsugae* (*GtMYB01-15*) (Supplementary Data S1). *G. boninense* clearly had more *MYB* genes than the other four species; this may be explained by its genome sequencing method, which used diploid material (https://www.ncbi.nlm.nih.gov/biosample?LinkName=bioproject_biosample_all&from_uid=421251). In *G. lucidum*, the 12 *MYB* genes were situated on seven chromosomes, with chromosome 3 containing the maximal number of three. Synteny analysis showed that 12 of the 13 *GsMYBs* distributed on the 9 scaffolds of *G. sinense* had a strong linear relationship with *GlMYB01-12* (Figure 1A). According to their numbers of repeat units, the 75 *Ganoderma MYB* genes were classified into four types: 1R-MYBs (52), 2R-MYBs (19), 3R-MYBs (2) and 4R-MYBs (2) (Supplementary Table S2). 1R-MYBs and 2R-MYBs existed in all five species, while 3R-MYBs were present in only *G. australe* (*GaMYB01*) and *G. lucidum* (*GlMYB07*), and 4R-MYBs were found in only *G. sinense* (*GsMYB01*) and *G. tsugae* (*GtMYB13*) (Figure 1B).

The lengths of the proteins encoded by these 75 *MYB* genes ranged from 192 (*GbMYB20*) to 1597 aa (*GaMYB07*) with an average of 705.24 aa. The calculated pI ranged from 4.59 (*GbMYB20*) to 11.01 (*GaMYB12*) with an average of 7.27, and the MW ranged from 21.42 (*GbMYB20*) to 171.92 (*GbMYB16*) kDa with an average of 77.00 kD. The GC content of the coding sequences ranged from 50.59 to 67.26%, with an average of 59.17%. A total of 56 *MYB* genes were localized to the nucleus, 11 to the cytosol and 19 to the mitochondria (Supplementary Table S2).

### Phylogenetic analysis of Ganoderma MYB genes

**Figure 2:**
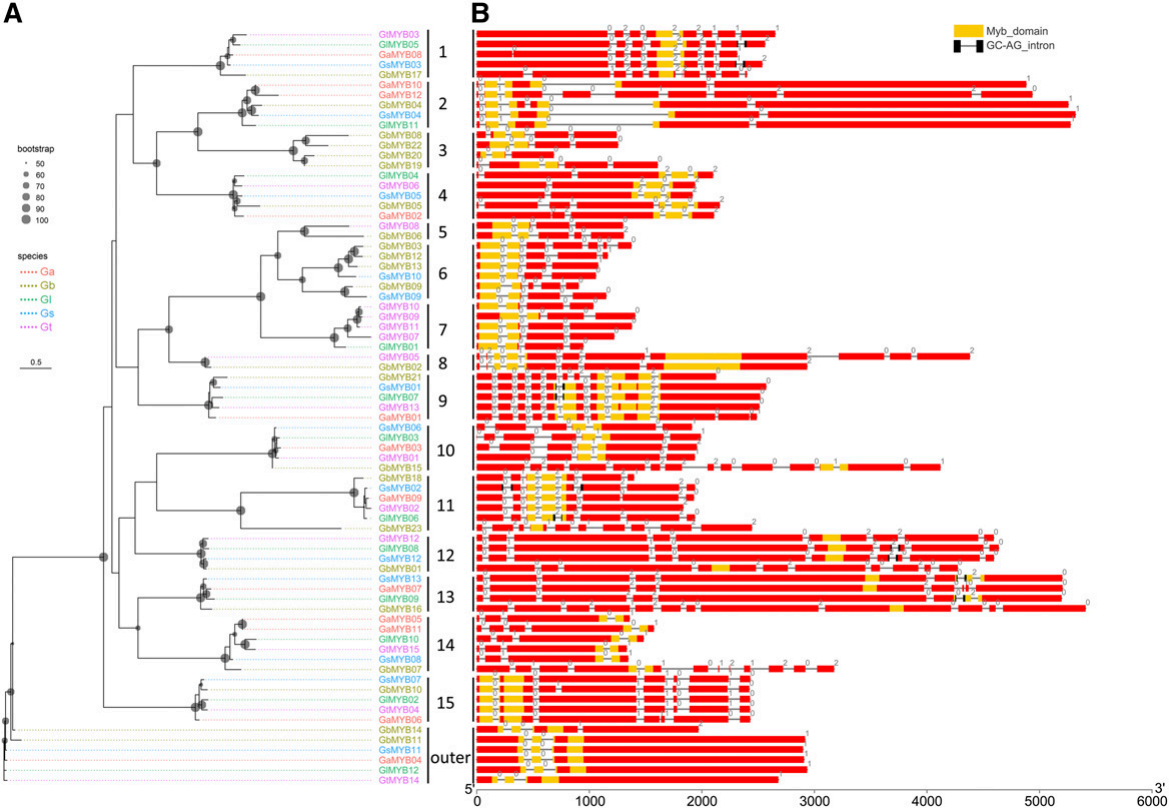
Phylogenetic relationships and gene structures of *Ganoderma MYB* genes. (A) The ML phylogenetic tree was based on full-length protein sequences with 1000 bootstrap resamplings; the dots on the nodes represent bootstrap values; Ga, *G. australe*; Gb, *G. boninense*; Gl, *G. lucidum*; Gs, *G. sinense*; Gt, *G. tsugae*; and the numbers behind the *MYB* genes represent the different subgroups. (B) Red rectangles and black lines represent exons and introns, respectively; the number above the black line represent the intron phase; the yellow rectangles represent MYB domains; and the black rectangles represent GC-AG introns.

To determine the phylogenetic and orthologous relationships among the MYB proteins, an ML phylogenetic tree was constructed using the full-length MYB protein sequences of the *Ganoderma* species, and orthologous groups were determined by OrthoMCL analysis (Supplementary Table S3). Sixty-nine *MYB* genes were divided into 15 subgroups (designated as subgroup 1 to subgroup 15), while the other 6 *MYB* genes (*GbMYB11*, *GbMYB14*, *GsMYB11*, *GaMYB04*, *GlMYB12* and *GtMYB14*) were designated as outer clade (Figure 2A). Different types of *MYB* genes tended to be classified into separate subgroups. Most subgroups (subgroups 1, 3, 4, 5, 6, 7, 10, 11, 12 and 14) contained 1R-MYBs only, subgroups 8 and 15 contained 2R-MYBs only, and subgroup 9 contained 2R-MYBs as well as all the 3R-MYBs and 4R-MYBs.

The *MYB* genes in subgroups 1, 4, 9, 10 and 15 were single-copy orthologous genes, because each of the *MYB* genes in a given subgroup had only one copy in each species. These *MYB* genes represented conserved sequences in *Ganoderma*, and may have originated from a common ancestor. On the other hand, intraspecific duplication events were found in *G. australe* and *G. tsugae*. In *G. australe*, the whole protein sequences of *GaMYB10* and *GaMYB12* showed an identity of 64.5%, and both the N-termini and C-termini had identical protein sequence patterns (Supplementary Figure S1); *GaMYB05* and *GaMYB11* showed a higher identity of 88.0%, and the difference was mainly in the extended N-terminus of 48 aa in *GaMYB11* (Supplementary Figure S2). In *G. tsugae*, *GtMYB10*, *GtMYB09*, *GtMYB11* and *GtMYB07* showed high mutual similarity and were grouped into subgroup 7, accompanied by a homolog, *GlMYB01* (Figure 2; Supplementary Figure S3).

In the phylogenetic analysis including *Ganoderma* and other organisms, the 75 *Ganoderma MYB* genes and 124 *MYB* genes from the other organisms formed multiple clades (Supplementary Table S1; Supplementary Figure S4). Clades consisting of *MYB* genes from different taxa may have evolved from the same ancestor and may have similar biological functions. Clades unique to *Ganoderma* also existed.

### Gene structure and MYB domain of Ganoderma MYB genes

**Figure 3:**
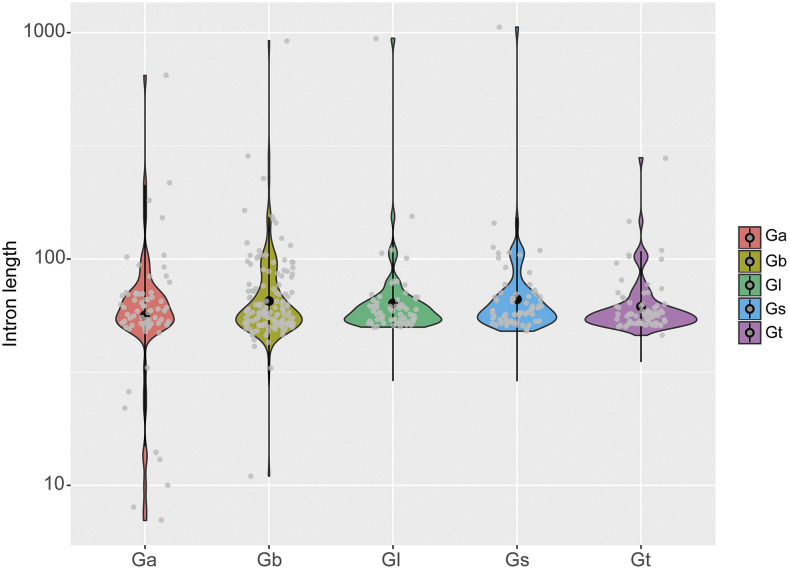
Distribution of intron length in *Ganoderma* species. The number represent log_10_(intron length); Ga, *G. australe*; Gb, *G. boninense*; Gl, *G. lucidum*; Gs, *G. sinense*; Gt, *G. tsugae*.

A rich diversity of *MYB* gene structure was observed in *Ganoderma*. Varying numbers of exons, from 3 to 14, and introns, from 2 to 13, were detected in the 75 *MYB* genes; intronless *MYB* genes were not found. In total, 489 exons and 414 introns were identified, and all the *MYB* genes started with 0-phase-introns. On average, the 1R-MYBs contained 5.48 introns, the 2R-MYBs contained 4.96 introns, the 3R-MYBs contained 9 introns, and the 4R-MYBs contained 8 introns. Two *MYB* genes had intron lengths shorter than 10 bp (*GaMYB02*, 7 bp; *GaMYB01*, 8 bp), while three had intron lengths of approximately 1000 bp (*GsMYB04*, 1058 bp; *GbMYB04*, 921 bp; *GlMYB11*, 944 bp). The average intron length was 74 bp across all five species, 71 bp in *G. australe*, 75 bp in *G. boninense*, 76 bp in *G. lucidum*, 81 bp in *G. sinense*, and 65 bp in *G. tsugae*. In general, most introns (90%) were 48-112 bp in length, and *G. tsugae* had most narrowest length distribution pattern, with all intron lengths between 46 and 280 bp (Figure 3; Supplementary Table S4).

Five GC-AG introns (noncanonical introns) in *G. lucidum* and six in *G. sinense* were detected, while most introns were classical GT-AG introns (Figure 2B). In *G. lucidum*, *GlMYB05* (1R-MYB), *GlMYB06* (1R-MYB), *GlMYB07* (3R-MYB), *GlMYB08* (1R-MYB) and *GlMYB09* (1R-MYB) each contained a single GC-AG intron (Figure 2B). In *G. sinense*, *GsMYB12* (1R-MYB) contained two GC-AG introns; *GsMYB01* (4R-MYB), *GsMYB02* (1R-MYB), *GsMYB03* (1R-MYB) and *GsMYB13* (2R-MYB) each contained one GC-AG intron (Figure 2B). The four gene pairs *GlMYB05*/*GsMYB03*, *GlMYB07*/*GsMYB01*, *GlMYB08*/*GsMYB12* and *GlMYB09*/*GsMYB13* each had identical gene structures (including exon number and intron phase) and the same GC-AG intron location, suggesting that the *MYB* genes containing GC-AG introns were conserved in *G. lucidum* and *G. sinense*.

In total, 104 MYB domains were identified in the 75 *MYB* genes, and most MYB domains were distributed in or near the N-terminus (Figure 2B). The MYB domains had a broad range of lengths, from 37 to 226 aa, with an average of 64.3 (Supplementary Table S5). The *MYB* genes in subgroups 12, 13 and 14 had clearly shorter MYB domains than those of other subgroups. Most MYB domains spanned the intron regions, indicating that they might be affected by alternative splicing. The regularly spaced tryptophans of the *MYB* domain were conserved within each subgroup yet diverse among subgroups (Supplementary Figure S5). In addition, each of the four *MYB* genes in subgroup 12 contained an HSA domain and *GbMYB15* contained a FAD_binding and a FAD-oxidase_C domain (Supplementary Table S6).

*MYB* genes in the same subgroup shared similar gene structures, including gene length, intron phase, number/location of exons and number/location/length of MYB domains. However, *MYB* genes in different subgroups showed significant differences. Subgroups 2, 12 and 13 contained obviously longer genes than the other groups, while subgroup 8 had an obviously longer MYB domain. These results show that the *MYB* gene structures were consistent with the phylogenetic topology.

### Promoter analysis of Ganoderma MYB genes

**Figure 4:**
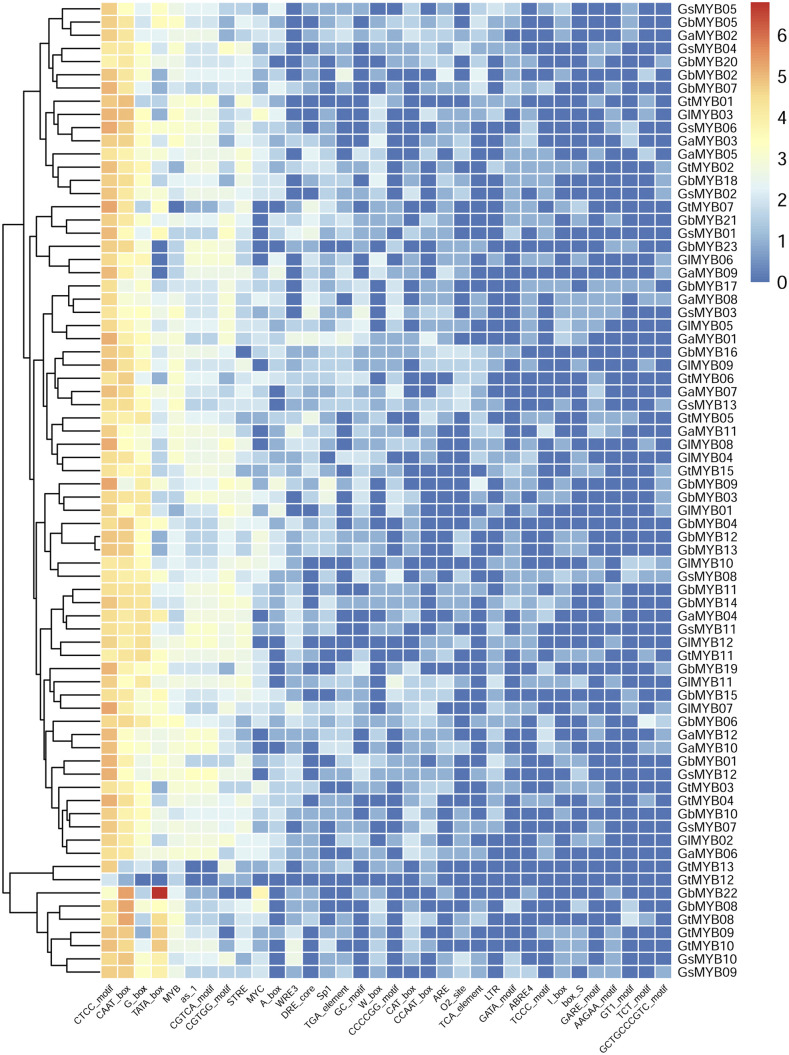
The 34 most frequent *cis*-elements in the MYB promoters. The color scale shown at the top represent log_2_(number of occurrences).

The promoter regions of the *Ganoderma MYB* genes had GC contents of 34.35–63.4%, with an average of 55.03% (Supplementary Table S2). The CTCC motif, CAAT box, G box and TATA box were the most frequently occurring *cis*-elements in the promoter regions. The CTCC motif and CAAT box occurred in all the promoters, with the CTCC motif having an average occurrence of 25.2 and the CAAT box having an average occurrence of 17.6 (Figure 4). Cis-elements related to light response (SP1, GATA motif, TCCC motif, GT1, and ATCT motif), stress response (STRE, MYC, LTR, and GC motif), hormone response (CGTCA motif, TGA, TCA element, and GARE motif) and developmental process (CAT box) (Skriver *et al.* 1991; Terzaghi & Cashmore 1995; Treger *et al.* 1998; Laloum *et al.* 2013; Zhang *et al.* 2016) existed in the promoters of some *MYB* genes, suggesting that these *MYB* genes may participate in multiple biological processes. Similar *cis*-element patterns were detected in the promoter regions of some duplicated gene pairs, such as *GaMYB10/12* and *GtMYB09/10* (Figure 4). However, different patterns were observed in other gene pairs, such as *GaMYB05/11* and *GtMYB07/11*, implying a diversity of potential functions among the duplicated *MYB* genes. Notably, MYB binding sites were identified in the promoters of all the detected *MYB* genes except *GtMYB07*, indicating extensive cooperation among *MYB* genes.

### Co-expression analysis of G. lucidum MYB genes

**Figure 5:**
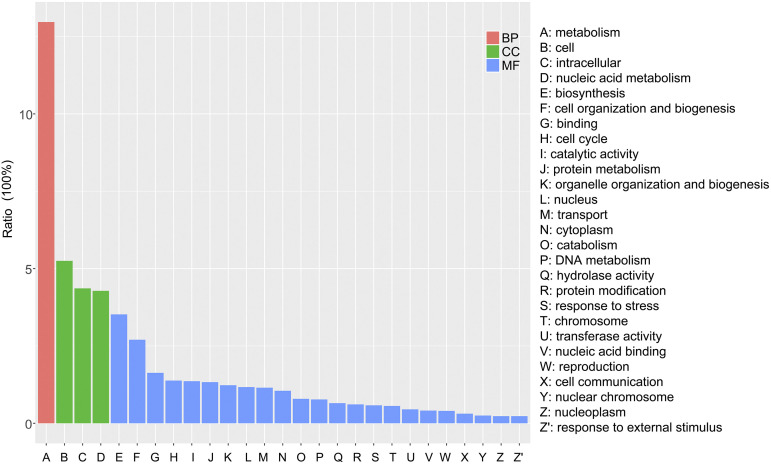
GO classifications of genes most related to *GlMYBs*. BP, biological process; CC, cellular component; MF, molecular function.

Based on the WGCNA analysis using 16 *G. lucidum* RNA-seq datasets from the NCBI database, 35 modules were identified, and 12 *GlMYBs* were clustered into 4 modules (Supplementary Figure S6). The genes that had the most similar expression patterns to the *GlMYBs* in the target module were classified into three main categories: biological process, cellular component and molecular function (Supplementary Data S2). The most enriched biological process (GO: 0008150) was metabolism (GO: 0008152). In the cellular component (GO: 0005575) category, cell (GO: 0005623), intracellular (GO: 0005622) and nucleic acid metabolism (GO: 0006139) were enriched. In the molecular function (GO: 0003674) category, biosynthesis (GO: 0009058), cell organization and biogenesis (GO: 0016043), binding (GO: 0005488), cell cycle (GO: 0007049), and catalytic activity (GO: 0003824) were the top five classes (Figure 5). These highly enriched GO terms were mainly relevant to cell metabolism, development, and regulation of gene expression, indicating functions for *GlMYBs* in related processes.

## Discussion

A genome-wide comprehensive analysis of *Ganoderma MYB* genes revealed both similarities and differences among *Ganoderma* species. The similarities are mainly reflected in three aspects: (1) 1R-MYBs and 2R-MYBs are general MYB types in *Ganoderma* species; (2) MYB orthologs that showed high similarity in the phylogenetic analysis might possess similar functions; and (3) CTCC motifs and CAAT boxes occur in all promoter regions with high frequency, indicating their conserved functions in the regulation of *MYB* genes. The differences are mainly reflected in four aspects: (1) the MYB number varies among different species, indicating gene gain and loss during speciation; (2) 3R-MYBs exist exclusively in *G. australe* and *G. lucidum*, and 4R-MYBs exist exclusively in *G. sinense* and *G. tsugae*; (3) GC-AG introns are observed in only *G. lucidum* and *G. sinense*; and (4) only some of the five species underwent *MYB* gene duplications. The divergence of *MYB* genes among species might contribute to species evolution and adaptability. Hence, we speculated that some *MYB* genes may be developed as markers for species identification.

The number of *MYB* gene family members varies greatly among species. The *MYB* gene number is dramatically expanded to 100-500 in plants (Chen *et al.* 2006; Salih *et al.* 2016; Yang *et al.* 2019; Li *et al.* 2020), with a reduced number of 4-5 in animals (Davidson *et al.* 2005) and a moderate number of 10-40 in fungi (Verma *et al.* 2017; Wang *et al.* 2018; Li *et al.* 2019). In this study, the *Ganoderma* species had 12 to 23 *MYB* genes, similar to other fungi. In addition, the dominant MYB type differs between fungi and plants. In plants, 2R-MYBs (R2R3) are the dominant MYB type, especially in *P. edulis*, which contains 96.47% 2R-MYBs (Yang *et al.* 2019). In addition, 2R-MYBs in plants tend to have conserved splicing patterns. For example, *A. thaliana* and rice have a 2R-MYB gene structure of three exons and two introns (Katiyar *et al.* 2012). However, in this study, the *Ganoderma* species had mostly 1R-MYBs, with no clearly conserved gene structure found. As the dominant 2R-MYBs play central roles in plant-specific processes (Lee & Seo 2019; He *et al.* 2020), it is likely that the dominant 1R-MYBs might play core roles in relevant processes in *Ganoderma* species.

Fungal introns are typically short, with mean intron lengths ranging from 69 bp in *Cryptococcus neoformans* to 256 bp in *Saccharomyces cerevisiae* (Kupfer *et al.* 2004). The average intron lengths in the whole gene sets of *G. lucidum* and *G. sinense* are 87 and 82 bp, respectively (Chen *et al.* 2012; Zhu *et al.* 2015). In this study, the average intron length of the *Ganoderma MYB* genes was 74 bp, consistent with the typical short introns of fungal species. However, three long introns with lengths of approximately 1000 bp were also identified. Studies have shown that gene expression can be impacted by intron length due to the increased time required to transcribe genes containing long introns (Swinburne *et al.* 2008). Intron length also determines the effect of the exon junction complex (Ashton-Beaucage *et al.* 2010), and genes containing long introns tend to have alternative splicing (Roy *et al.* 2008). However, the possible functions of the long introns in the *Ganoderma MYB* genes need to be elucidated further.

In total, 10 of the 75 *MYB* genes were found to contain GC-AG introns, and these *MYB* genes may recruit different splicing mechanisms during mRNA maturation. Studies have shown that 98% or more of introns are canonically spliced (GT-AG) in fungi, and GC-AG introns usually have an occurrence of approximately 1% (Kupfer *et al.* 2004). In *G. lucidum* and *G. sinense*, 41.7% and 38.5% of the *MYB* genes contained GC-AG introns, indicating a high proportion of noncanonical introns in *Ganoderma MYB* genes. More sophisticated splicing mechanisms are active in *G. lucidum* and *G. sinense*, and the significance of these GC-AG introns needs further study. Moreover, this reminds us that the traditional gene prediction method could be unconvinced without consideration of noncanonical splicing.

The analysis of the *cis*-elements in the promoter regions of *Ganoderma MYB* genes and the co-expressed target genes of the *GlMYBs* help us understand the potential functions of *MYB* genes in *Ganoderma*. Similar to the roles *MYB* genes play in plants, *Ganoderma MYB* genes are widely involved in various biological processes, including stress response, development, and metabolism. Given their important regulatory roles, *MYB* genes should be included in future studies of macrofungal biological processes.

## Conclusion

In this study, a total of 75 *MYB* genes were identified in five *Ganoderma* species, of which 69 were clustered into 15 subgroups. Both single-copy orthologous genes and duplicated genes were identified in subgroups. Varying sequence characteristics were observed among subgroups. Multiple regulatory *cis*-elements existed in the promoters of *Ganoderma MYB* genes, and some genes related to stress response, development and metabolism co-expressed with *GlMYBs*. Our results suggest that *MYB* genes participate in multiple biological processes in *Ganoderma*. However, further function validation is required in the future. Given the increasing interest in *Ganoderma* species, the fundamental information of *MYB* genes from this study will facilitate the biological studies in this genus.
